# Artificial intelligence-based non-small cell lung cancer transcriptome RNA-sequence analysis technology selection guide

**DOI:** 10.3389/fbioe.2023.1081950

**Published:** 2023-02-15

**Authors:** Min Soo Joo, Kyoung-Ho Pyo, Jong-Moon Chung, Byoung Chul Cho

**Affiliations:** ^1^ School of Electrical and Electronic Engineering, College of Engineering, Yonsei University, Seoul, Republic of Korea; ^2^ Department of Oncology, Severance Hospital, College of Medicine, Yonsei University, Seoul, Republic of Korea; ^3^ Severance Biomedical Science Institute, Yonsei University College of Medicine, Seoul, Republic of Korea; ^4^ Yonsei New Il Han Institute for Integrative Lung Cancer Research, Yonsei University College of Medicine, Seoul, Republic of Korea; ^5^ Division of Medical Oncology, Department of Internal Medicine and Yonsei Cancer Center, Severance Hospital, Yonsei University College of Medicine, Seoul, Republic of Korea; ^6^ Department of Emergency Medicine, College of Medicine, Yonsei University, Seoul, Republic of Korea

**Keywords:** non-small cell lung cancer, transcriptome, RNA, sequence, statistical analysis, machine learning, deep learning

## Abstract

The incidence and mortality rates of lung cancer are high worldwide, where non-small cell lung cancer (NSCLC) accounts for more than 85% of lung cancer cases. Recent non-small cell lung cancer research has been focused on analyzing patient prognosis after surgery and identifying mechanisms in connection with clinical cohort and ribonucleic acid (RNA) sequencing data, including single-cell ribonucleic acid (scRNA) sequencing data. This paper investigates statistical techniques and artificial intelligence (AI) based non-small cell lung cancer transcriptome data analysis methods divided into target and analysis technology groups. The methodologies of transcriptome data were schematically categorized so researchers can easily match analysis methods according to their goals. The most widely known and frequently utilized transcriptome analysis goal is to find essential biomarkers and classify carcinomas and cluster NSCLC subtypes. Transcriptome analysis methods are divided into three major categories: Statistical analysis, machine learning, and deep learning. Specific models and ensemble techniques typically used in NSCLC analysis are summarized in this paper, with the intent to lay a foundation for advanced research by converging and linking the various analysis methods available.

## 1 Introduction

According to data published in the Cancer Journal for Clinicians (Sung et al., 2021), the number of cancer patients worldwide has increased from 10 million in 2000 to 19.3 million in 2020. During this 20 years period, the number of incidences of lung cancer was the undisputed number one type of cancer. The number of lung cancer patients in 2021 ranked second with 22 million, or 11.4% of all cancer patients. However, as lung cancer ranks first in mortality, it is a disease that has a great impact on human society. Accordingly, various methods of research are being conducted worldwide to elucidate growth mechanisms of lung cancer and develop effective therapeutic agents. Research on non-small cell lung cancer (NSCLC) is predominant among lung cancer types, as it accounts for 80 ∼85% of all lung cancer incidents.

In this paper, various artificial intelligence (AI) based transcriptome analysis methods and predictive models are investigated to provide future guidelines for more effective NSCLC treatment development. NSCLC can be subdivided into squamous cell carcinoma, adenocarcinoma, and large cell carcinoma, among which squamous cell carcinoma and adenocarcinoma are most common. Mutations are another important element in the study of NSCLC and being able to accurately classify lung cancer types is essential in selecting treatment options and identifying oncologic mechanisms. This is because the targeted therapy or chemotherapy applied to lung adenocarcinoma (LUAD) and lung squamous cell carcinoma (LUSC) patients are different (Coudray et al., 2018). In addition to the analysis method of classifying lung cancer subtypes, detailed classification of lung cancer mutations can also elucidate mechanisms and help derive the most effective biomarkers (Zhao et al., 2015; Cohen et al., 2020).

These studies are based on next-generation sequencing (NGS) analysis (De Luca et al., 2021). As research to elucidate these molecular biological mechanisms has been actively conducted, various diagnostic methods and new targeted therapies have become possible. With the development of NGS technology, various genetic mutations have been reported in patients with NSCLC. However, in actual clinical practice, the test methods to determine the treatment policy for NSCLC patients are limited to epidermal growth factor receptor (EGFR) gene mutations and anaplastic lymphoma kinase (ALK) fusion genes (Soda et al., 2007; Hida et al., 2017). Targeted therapy has a high need for whole exome sequencing (WES) and whole genome sequencing (WGS), which can explain the mechanism of resistance for targeted therapies (Kruglyak et al., 2016). However, in the case of immune-therapeutic agents that have been developed, analysis of the expression and pattern of immune-related genes has become more important than mutations secured through genome-wide analysis, so research on translation analysis has emerged.

In recent years, research that enables direct clinical application by applying a cancer prognosis (Han et al., 2019; Volckmar et al., 2019), metastasis (Qi et al., 2017; Kamer et al., 2020; Kim et al., 2020; Tao et al., 2020), and/or treatment response (Jiang et al., 2018) prediction model has been in the spotlight. A representative example is a research method in which a specific overexpressed RNA is discovered and selected to be used as a targeted therapeutic agent in reference to the nature of cancer, in which gene mutation is the main etiology. Although the details will be described later, most studies aim at discovering biomarkers to determine the overall survival (OS) as the output (Yuan et al., 2017; Givechian et al., 2019; Xiong et al., 2020). Although there are limits to accurate analysis of tens of thousands of features per patient using various techniques, this is the most widely used method in modern RNA-seq analysis research.

To help the RNA-seqeuncing (RNA-seq) analysis process, this paper focuses on providing a guideline on various AI and mathematical and statistical methods that can be used in extracting effective RNA information and discovering biomarkers for NSCLC patients. Various RNA-seq analysis methods, such as gene expression, gene fusion, and mutation, have been applied in the past. In addition, gene set enrichment analysis (GSEA) and pathway analysis have been used in various medical fields to extract gene expressions. The accuracy of the prognosis or symptom to be predicted varies depending on the AI model used, and many approaches already exist, which are difficult to distinguish the pros and cons. Therefore, this paper investigates various RNA-seq analysis methods using AI technologies in hope to help future clinical research. For example, a binary prediction model that classifies and clusters a patient’s tumor mutation from raw data of the RNA-seq can be constructed, or a regression model that predicts whether a patient will recur or not can be designed.

In this paper, RNA-seq analysis using AI technologies are divided into three categories, which are statistical analysis, machine learning, and deep learning. Statistical analysis is effective in finding out which gene groups have significant values. Statistical techniques help to visualize the analyzed result as a volcano plot or heatmap to identify the tendency. Various test techniques are included by applying existing statistical analysis methods used in other fields (Sun et al., 2018; Passiglia et al., 2019). The regression method is represented by the Cox Proportional Hazard model (Cox-ph model) approach (Sun et al., 2022). Survival analysis based on the Cox-ph model are predominant. Tools such as TIDE is a representative example (Jiang et al., 2018). The cox-ph model is a method extensively and generously used in NSCLC RNA-seq analysis and is classified as a statistical analysis category because it is standardized with a specific formula. Machine learning techniques are mainly used for classification and prediction analysis. For example, subtypes of NSCLC can be distinguished using SVM, or tumor mutations can be clustered using logistic regression. Among advanced machine learning techniques, representative schemes include supervised learning and AutoEncoder with Cox regression network (AECOX) (Huang et al., 2020), which discovers specific expression genes by combining models (e.g., SVM and universal classification tools) to construct an ensembled-model, and methods combining two or more machine learning tools are under development. Deep learning-based analysis is mainly used as a method of substituting a neural network into a pipeline. For example, Cox-nnet (Travers et al., 2018) inserts a neural network in front of the Cox-ph model, and DeepSurv (Katzman et al., 2018) uses a neural network in the survival analysis. DCNet (Wang et al., 2022) is a good model to classify subtypes of lung cancer.

## 2 RNA-sequencing analysis identified by target

### 2.1 Preprocessing data for analysis

**FIGURE 1:**
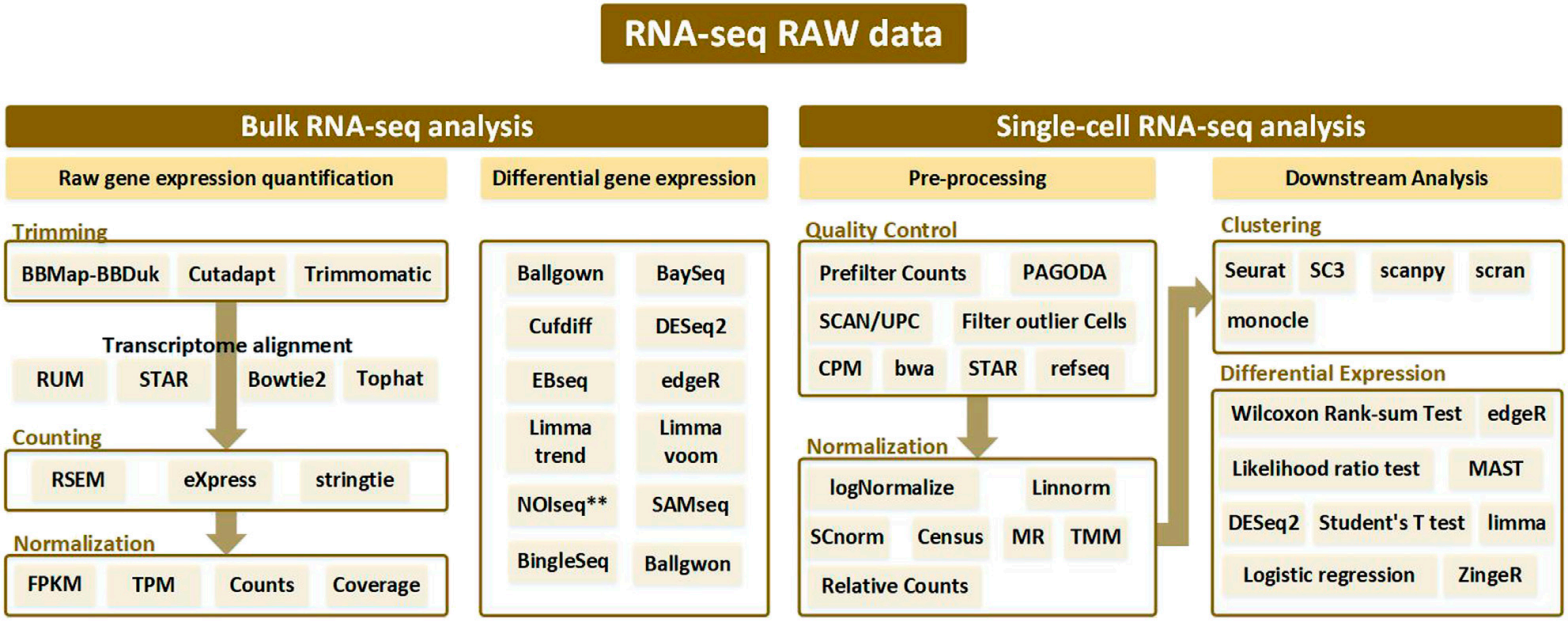
Analysis pipeline according to bulk RNA-seq and single-cell RNA-seq from RNA-seq raw data. Bulk RNA-seq analysis is divided into two pipelines: raw gene expression quantification (which leads to trimming, counting, and normalization) and differential gene expression analysis. Alignment is performed after trimming to remove contamination from the sequenced data, and various tools used at this time are generally bowtie-based. Afterwards, the counting process is performed to remove the biased sequencing data, and after normalization, the processed data goes through the final step of discriminating the expressed gene through differential gene expression analysis. In single-cell RNA-seq analysis, processes such as trimming and mapping are batch-processed in the quality control stage, and normalization is performed. After that, a clustering procedure to differentiate between similar cell types is essential. Then the single-cell RNA-seq analysis workflow is completed with differential expression analysis.

**FIGURE 2:**
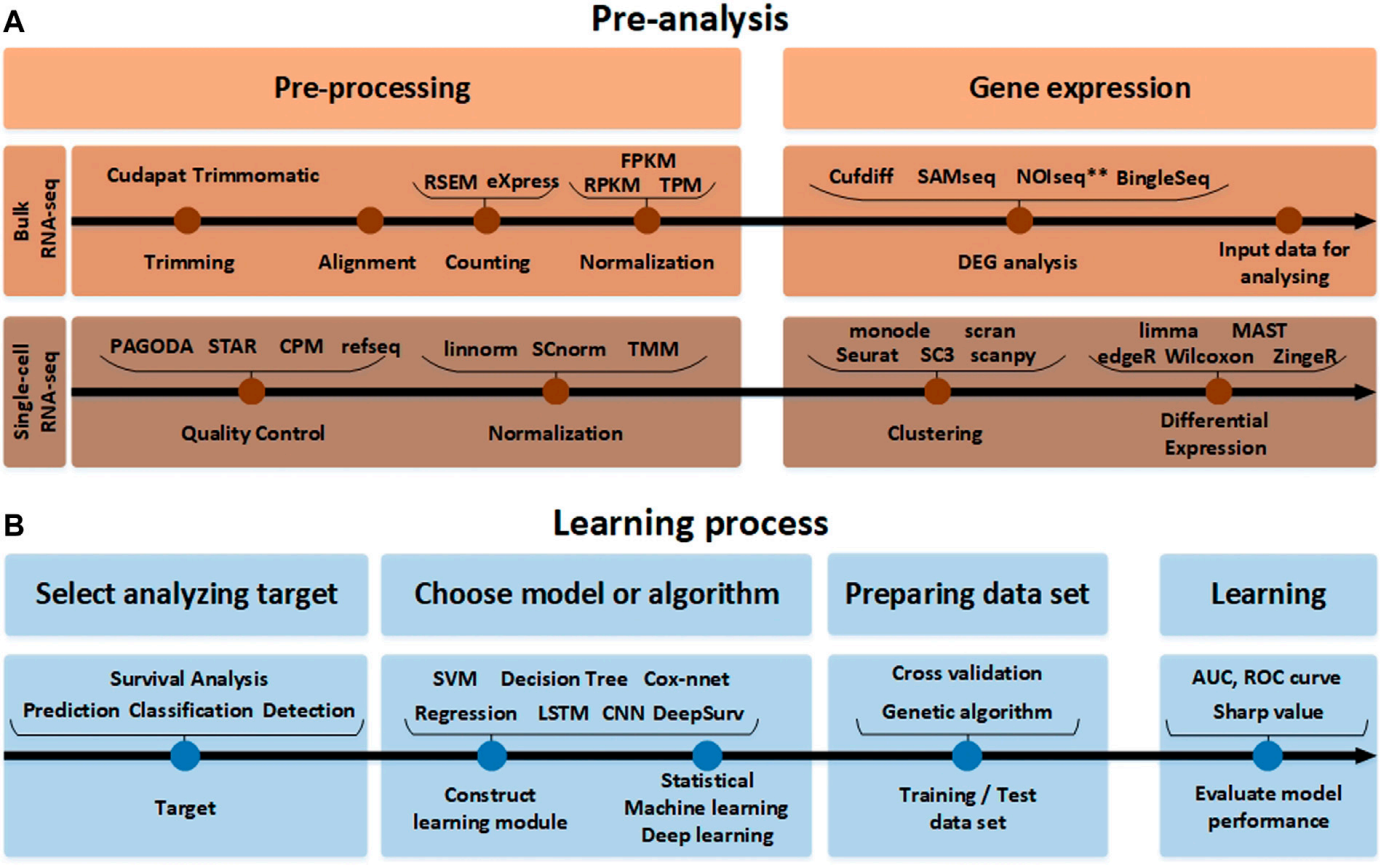
The RNA-seq data analysis process. **(A)** Before the analysis of RNA-seq data. The pre-analysis part includes the conversion process to form the input data into an appropriate format that can help in analyzing the RNA-seq data. Since the detailed tools used for processing and gene expression analysis are different in single-cell RNA-seq and bulk RNA-seq analysis methods, they are indicated separately. **(B)** The learning process part after the analysis of RNA-seq data. In accordance with the target to analyze the input data obtained in this way. It it beneficial to pre-process the data using various normalization tools again and learn through the AI model.

To properly use RNA-seq data, preprocessing is indispensable, as the range of values varies according to the type of RNA-seq and because gene expressions are different depending on the patient and patient group. The order of the preprocessing RNA-seq data method is slightly different depending on the nature of the RNA-seq data. First, in the case of bulk RNA-seq data, differentially expressed genes are derived through trimming, counting, and normalization. An alignment process may be added after trimming, which is an additional way to align data. Second, in the case of single-cell RNA-seq data, Quality Control and Normalization are performed, and the results are grouped through Clustering. After this, differential expressed genes were obtained in each group. For more details on the process, please refer to Figure 2B and Figure 6. Gene selection plays a role in reducing the absolute amount of RNA-seq data to be analyzed by extracting a specifically expressed gene mainly through machine learning. Gene selection creates an environment in which bio-marker discovery can be made easier by reducing the analysis time and deriving more effective factors. In particular, genes are selected through a classifier, such as a decision tree or support vector machine (SVM). As shown in Figure 1, by using the normalization technique, which is often used in statistics, it is possible to unify the data units and reduce the influence of the size factor. In addition, using the ranking expression technique, it is possible to determine the criteria for an appropriate data set by obtaining a differential expression. Through this, the number of genes can be adjusted according to the analysis goal and used for further detailed analysis. Survival analysis is commonly conducted using traditional statistical analysis methods rather than AI techniques. Prognostic analysis uses various learning methods from machine learning and deep learning, where recently, tumor microenvironment analysis applying convolutional neural network (CNN) technology using image data is being actively conducted.

### 2.2 Categorization of RNA-sequencing analysis techniques

Research using RNA-seq can be subdivided according to various purposes. Depending on the target, it is broadly classified into classification and prediction in a wide range, but it can be subdivided into biomarker, detection, survival analysis, *etc.* In the predictive biomarker category, studies were also conducted to identify immune checkpoint inhibitors (ICB) (Wiesweg et al., 2019) or to identify the mechanisms of biomarkers that affect responsiveness to immunotherapy (Jiang et al., 2018). Beyond simple elucidation of biomarkers, technologies can be further subdivided based on research that analyzes the prognosis of responsiveness to immunotherapy (Auslander et al., 2018; Jiang et al., 2018; Kapil et al., 2018; Althammer et al., 2019; Nikolas Kather et al., 2019; Fu et al., 2020; He et al., 2020). There are also analysis methods that predict metastasis (Qi et al., 2017; Kamer et al., 2020; Kim et al., 2020; Tao et al., 2020) or identifies indicators related to recurrence after cure or treatment (Lu et al., 2012; Galvez et al., 2020). Analysis of mutation patterns in lung cancer (Zhao et al., 2015; Cohen et al., 2020) and prognostic prediction (Han et al., 2019; Volckmar et al., 2019), which predicts the prognosis of lung cancer patients differently from the previous prediction category, are analyzed as targets, and survival analysis (Yuan et al., 2017; Givechian et al., 2019; Xiong et al., 2020) can be classified as a prognostic biomarker category. Subtypes of lung cancer can be classified by applying ensemble machine learning tools with multi-class classification capability. The process starts with an analysis (Huang et al., 2017; Hsu and Dong, 2018) that classifies malignant and benign based binary classification using various machine learning techniques (Cai et al., 2015; Tian, 2017; Su et al., 2020; Huang et al., 2021; Wang et al., 2022).

In connection with cancer classification analysis, AI techniques are used to classify the reactive prognosis according to the type of surgery or the patient group that distinguishes between malignant and benign status. In this case, since there are two final analysis targets, machine learning tools such as SVM or decision tree are suitable to use as they are very effective in binary classification (Han et al., 2014; Peng et al., 2015; Huang et al., 2017; Hsu and Dong, 2018; Reynders et al., 2018). In addition, analysis based on gender, age, overall survival (OS), *etc.*, is also needed. In general, classifying cancer subtypes of NSCLC is treated as a multi-group classification problem because there are more than three groups to be distinguished (Cai et al., 2015; Tian, 2017; Su et al., 2020).

Prediction techniques are also very important as they help estimate future prognosis based on surgery or treatment method (Zhou et al., 2016; Ahmed et al., 2018; Han et al., 2019; Volckmar et al., 2019; He et al., 2020). In addition, progression-free survival (PFS) analysis has been used since the 2010s to predict the recurrence probability (Lu et al., 2012; Galvez et al., 2020). It is also possible to rank treated patients by extracting expressed genes and analyzing RNA based on how highly a specific treatment was responsive (Han et al., 2019). Studies that have focused on survival analysis use various criteria ranging from simply distinguishing between dead and alive (Jefferson et al., 1997) to predicting the survival rate (Yuan et al., 2017; Givechian et al., 2019; Xiong et al., 2020). Among these studies, RNA-seq data has been used in survival analysis in many ways, which are based on the various targets of classification. Analysis using only numerical values of the RNA from a statistical point of view has been performed by various researchers (Li et al., 2014; Conesa et al., 2016; Afonso et al., 2019; Sharma et al., 2019). In (Byron et al., 2016), the authors only use RNA-seq data in making survival predictions, where the data was transformed to fit the format of clinical data.

### 2.3 Process of RNA-sequencing data analysis

After setting the analysis target, the overall process flow is explained in the following. First, it is critical to pre-process the data in accordance with the analysis module and decide if clinical data will be combined. If the goal is to analyze a patient’s prognosis, adding clinical data increases the accuracy. On the other hand, a simple classification process that distinguishes between malignant and benign NSCLC patient predictions can be conducted without clinical data. Therefore, it is important to add or subtract data according to the analysis target. Once the data is ready, it should go through a normalization process. This process facilitates gene expression analysis because each RNA has a different scale. For example, the RNA normalization process includes fragments per kilobase of transcript per million mapped reads (FPKM), reads per kilobase per millions mapped reads (RPKM), and transcripts per million (TPM). However, if the extracted RNA-seq data does not show a significant difference in scale, the raw counts of RNA data itself can be directly used.

#### 2.3.1 Analysis pipeline of bulk RNA-sequencing

This procedure is a preparation step for analysis using FASTQ from raw RNA-seq data. FASTQ is a text-based format to store all quality (Phred) scores for each nucleotide expressed in ASCII code in the adenine, guanine, cytosine, thymine (AGCT) biological sequence. It can be divided into a technique to quantify and analyze gene expression from RNA-seq raw data and a technique to analyze differential gene expressions. First, considering the gene expression quantification technique, it mainly proceeds to 1) Trimming, 2) Counting, and 3) Normalization. Trimming is the procedure of removing contaminated or low-quality data from raw data. It is an optional step, but the quality of the data increases when it is executed, and the final analysis accuracy increases. Differentially expressed genes (DEG) analysis is a process of extracting meaningful RNAs. For this purpose, various test techniques can be used based on the *p*-value and the fold change (FC) value. DEG analysis can be performed with the Kaplan-Meier test using the log-rank test, or DEG analysis can be performed through gene pathway analysis. As shown in Figure 2A, the input data to be applied to the AI module needs to be prepared through this process. The next step is to build a learning module to apply the input data based on the analysis target, which can be assisted by the methods presented in Table 1 and Table 2. In AI analysis, the ratio of dividing the data for training, testing, and validation are important. In general, the analysis performed is good when the data is divided in a balanced form among the training and test sets, and it is useful to use validation techniques such as cross-validation. However, in addition to various validation methods and test set split methods, genetic algorithm-based approaches also exist, which are effective when the data is unbalanced. Genetic algorithms help in selecting several solutions in advance and obtains the most suitable solution over generations. If a genetic algorithm is applied to spilt the data, the genetic algorithm can be used to appropriately divide the training data set and the test data set so artificial intelligence technologies can be effectively applied. When there is abundant patient data, this is less of a problem, but in most cases, patient data is limited, and the ratio of dividing the training, validation, and test set is dependent on the data features and AI analysis algorithm.

**TABLE 1:** Analysis methodology identified by target. Three categories of analysis methodology are identified based on by the target: analysis methods that identifies a predictive biomarker according to the target of the analysis, identify biomarkers related to the prognosis of the patient, and classify the type of cancer.

Category	Target	Author and year of publication
Predictive Biomarker	ICB	Wiesweg et al. (2019)
	Response to Immunotherapy	Jiang et al. (2018)
	Metastasis	Qi et al. (2017)
		Kim et al. (2020)
		Kamer et al. (2020)
		Tao et al. (2020)
	Recurrence	Lu et al. (2012)
		Galvez et al. (2020)
Prognostic Biomarker	Mutation	Cohen et al. (2020)
		Zhao et al. (2015)
	Prognostic prediction	Han et al. (2019)
		Volckmar et al. (2019)
	Survival Analysis	Yuan et al. (2017)
		Givechian et al. (2019)
		Xiong et al. (2020)
Cancer Classification	Malignant or Benign	Hsu and Dong (2018)
		Huang et al. (2017)
	Cancer subtype	Tian (2017)
		Su et al. (2020)
		Cai et al. (2015)
		Huang et al. (2021)
		Wang et al. (2022)

**TABLE 2:** Analysis methodology identified by AI technique. The statistical part includes analysis using simple mathematical models including test techniques and regression represented by the Cox-ph model. AI techniques using big data can be divided into machine learning and deep learning. In the machine learning part, SVM is mainly used, and the ensembled model with Decision Tree added is also increasing in frequency recently. In the Deep Learning part, research is actively underway by adding neural networks to various models.

Category	Model & algorithm	Author and year of publication
Statistical Analysis	Copy Number Variant (CNV)	Zhao et al. (2015)
	Kaplan-Meier, Log-rank Test	Sun et al. (2018)
	Gene-Expression	Lu et al. (2012)
	Cox-ph model	Sun et al. (2022)
	TIDE	Jiang et al. (2018)
Machine Learning	Ensembled-model	Wiesweg et al. (2019)
	SVM	Zhou et al. (2016)
		Cai et al. (2015)
		Su et al. (2020)
	LR	Tian (2017)
		Cai et al. (2015)
	WGRFE	Su et al. (2020)
	AECOX	Huang et al. (2020)
Deep Learning	Neural Network	Faraggi and Simon (1995)
		Travers et al. (2018)
	DeepSurv	Katzman et al. (2018)
		Jiang et al. (2020)
		Huang et al. (2021)
		Wang et al. (2022)

After completing the previous three steps of Figure 2B, in the final performance evaluation process, the performance of the area under the curve (AUC) and receiver operating characteristic (ROC) curves need to be analyzed. Or the ranking of RNA expressions through the Sharpe value needs to be analyzed. These processes are summarized in Figure 2 and Table 1. Through the analysis that actively utilizes Table 2, the entire process of RNA-seq data analysis of NSCLC patients can be confirmed.

#### 2.3.2 Analysis pipeline of single-cell RNA-sequencing

Until now, the transcriptome of a cell population has been studied, but the precise results of the study are limited because the expression patterns of each cell are different. Therefore, there is a limit to the analysis to understand the cancer microenvironment in which various types of cells exist only by bulk RNA-seq. A method for deconvolution of the cell type ratio was developed from the bulk RNA-seq results (Sun et al., 2022). But since this method requires significant reference data, this increases the complexity even more, and therefore may need to be verified in another way, which may be difficult in reality. Accordingly, there was a demand to study cell interactions and tissue functions through cell-level analysis, and as a part of this, research on single-cell RNA-seq emerged. Single-cell RNA-seq analysis can be largely divided into a pre-processing part and a downstream analysis part. In the pre-processing part, quality control is performed first. Quality control in raw read is tested through various methods to confirm sequencing fish, PCR artifacts, and contamination. This is an essential step to remove outliers. In simple terms, it can be said that it is the task of selecting data for easy analysis. Data that has undergone quality control goes through normalization, and then the pre-processing (for analysis) is finished. Then by clustering similar references, differential expression analysis can be performed. The data after this process is now ready to be applied to machine learning tools.

## 3 RNA-sequencing analysis using artificial intelligence techniques

RNA-seq analysis methods can be classified based on the data type to be analyzed and the AI technique to be applied. Analysis methods using AI can be divided into three categories: statistical analysis, machine learning, and deep learning. First, statistical analysis methods can be divided into test technique, proportional hazard model, and regression. Test techniques can be subdivided into the Kaplan-Meier test (Fu et al., 2020) and log-Rank test (Sun et al., 2018), which are frequently used in survival prediction and deriving gene expressions (Lu et al., 2012). A more advanced analysis method is the regression method represented by the Cox-ph model. Survival analysis based on the Cox-ph model has shown a predominant performance, in which TIDE is a representative example (Jiang et al., 2018). Statistical analysis techniques are advantageous when the data set is small or when the target to be analyzed is clear. For example, it is effective to use statistical analysis when identifying the tendency in the presence or absence of cancer recurrence. Second, in machine learning, supervised learning and AECOX (Huang et al., 2020), which discovers specific expression genes by combining models (e.g., SVM), universal classification tools (to construct ensembled-models), and methods combining two or more machine learning tools have been developed. Machine learning techniques such as SVM, random forest and decision trees are mainly used for classification and prediction, which help distinguish the subtype of the NSCLC or the normal and abnormal status. Machine learning algorithms show a good performance in binary classification (Han et al., 2014; Peng et al., 2015; Huang et al., 2017; Hsu and Dong, 2018; Reynders et al., 2018) and multi-class classification (Tian, 2017; Su et al., 2020), where the analysis method differs depending on the nature of the input data. Depending on the raw-data and target classification domain, there are various methods that can be applied from basic machine learning techniques, which include SVM, logistic regression, artificial neural network (ANN) (Khan et al., 2001), and AECOX (Huang et al., 2020), which combines neural networks. Third, deep learning is a category of machine learning that uses ANNs with multiple hidden layers (and each hidden layer consists of more artificial neurons) to provide higher levels of precision, classification, and estimations. Deep learning has been used in AECOX models and regression models like Cox-nnet (Travers et al., 2018). In case the data set is small, deep transfer learning can be applied, or a pre-trained model can be imported and used in the analysis (Jiang et al., 2020). In the following, an overview of AI models used in NSCLC research is provided.

### 3.1 Statistical analysis and simple regression

Medical practitioners who study cancer patients as well as NSCLC mainly use the survival model. It is a statistical analysis method that estimates the survival time from the start of treatment to death of a patient (Kapil et al., 2018; He et al., 2020). However, since it focuses on simple ‘death,’ it is an analytical method that has limitations in being able to accurately analyze causal relationships. Regression techniques include the Kaplan-Meier test, log-Rank test, and the Cox’s proportional hazard (Cox-ph) model. The Kaplan-Meier test has a disadvantage in that it cannot control its variables, whereas the Cox-ph model enables variable control and is therefore most frequently used. Among regression methods, Kaplan-Meier (Fu et al., 2020), log-rank test (Auslander et al., 2018; Fu et al., 2020), and Cox-ph are most frequently used, so they are briefly described in the following. Most regression analysis algorithms use standard methods (e.g., *p*-value), where it is possible to cluster patients based on a major classification criterion. For example, regression is commonly used to divide the overall survival rate, disease-free survival rate, and median survival value into groups of patients who have undergone different treatment methods and determine the significance of the survival rate between the two groups.

#### 3.1.1 Kaplan-Meier

In (Qi et al., 2017), the Kaplan-Meier method was used based on the time of lung cancer surgery as the starting point to analyze the prognosis of patients who underwent surgery for metastatic lung cancer. The Kalman-Meier method (which is also called the product limit method) is effective in calculating the interval survival rate (at each event point during the entire study or analysis period) and finally calculates the cumulative survival rate. Applying this method to the RNA-seq data set, the survival rate can be calculated based on the occurrence of cancer. After that, the data can be arranged in the order in which cancer patients were observed, and the interval survival rate *P*(*k*) can be calculated based on the ratio of the number of survivors in each interval. For example, if one person dies during the observation period, the interval survival rate is (*n* − 1)/*n*, where *n* is the number of patients under observation. Finally, the cumulative survival rate *S*(*k*) required for the Kalman-Meier test can be obtained by sequentially multiplying the interval survival rate according to Eq. 1, where *N*_*s*_ is the number of survivors up to period *k* and *N*_*o*_ represents the number of observations up to period *k*

$$\;P\left(k\right)=\frac{N_{S}}{N_{O}}$$ (1)

#### 3.1.2 Log-rank test

The Kaplan-Meier test is an effective method for estimating the interval survival rate and cumulative survival rate, the log-rank test is effective in checking whether the difference in the survival rate between two groups to be discriminated is significant. For example, after using the Kaplan-Meier method, the log-rank test (applying the criteria of *p*-value less than 0.05) is used to compare the survival rates according to the disease-free survival period in (Qi et al., 2017). After the survival curve is drawn using the Kaplan-Meier method, the log-rank test plays a role in determining whether the groups have a significant statistical difference. The log-rank test is most effective when it is simply not possible to visually distinguish differences in survival curves. For example, in some cases a group that appears to have a relatively low survival rate may actually have a high survival rate, which can be accurately confirmed using the log-rank statistical test method.

#### 3.1.3 Cox Proportional Hazard model

The Cox-ph model is the best-known method for screening prognostic variables that have a significant effect on the survival rate of lung cancer. In particular, the Cox-ph model is commonly used to compare patients with any RNA factor in two patient groups. For example, a group of patients with a high and low TGFB1 ratio has been analyzed as a survival fraction over time in (Cohen et al., 2020). A new biomarker can be estimated by selecting significant RNA groups among multiple RNA groups using the Cox-ph model. Most RNA sequence studies aim to calculate a significant biomarker candidate group by using this proportional hazard model and set a standard using the built-in *p*-value and fold change value. The previous Kaplan-Meier test and log-rank test are non-parametric analysis methods because they do not reflect the characteristics of the data. On the other hand, the Cox-ph model can predict the survival period using a regression model under the assumption that the survival time distribution (e.g., normal distribution) of lung cancer patients exists. In addition, the Cox-ph model utilizes the hazard ratio (HR), where it assumes that the HR is always constant. The Cox-ph model uses survival functions like Kaplan-Meier, where a survival function expressed by *S*(*t*) represents 1 at first, and *S*(*t*) would converge to 0 when infinite time has elapsed. Using a NSCLC patient as an example, the Cox-ph model will have *S*(*t*) = 1 at the starting point of observation. Through the survival function, it is possible to derive the lifetime distribution function *F*(*t*), which represents the probability that the observed event will occur within a specific time. By differentiating the lifetime distribution function, it is possible to obtain the survival density function *f*(*t*), which represents the event rate at a specific time. The relation of *S*(*t*), *F*(*t*), and *f*(*t*) is expressed in Eq. 2.

$$\;S\left(t\right)=P\left(T>t\right)=1-F\left(t\right)=\int_{0}^{\infty }f\left(u\right)du$$ (2)

The hazard function *H*(*t*) uses the probability that an event will occur at an arbitrary point in time *h*(*t*). The hazard function is based on a conditional probability that represents the probability that an event will occur for a case in which the event has not occurred until a specific time. That is, the hazard function *H*(*t*) uses the probability relation of *h*(*t*) = *f*(*t*)/*s*(*t*) and is defined in Eq. 3.

$$\;H\left(t\right)=\int_{0}^{t}h\left(u\right)du=-logS\left(t\right)$$ (3)

### 3.2 Machine learning

In the analysis of RNA sequencing of NSCLC using machine learning, supervised learning techniques represented by SVM classify subtypes of cancer, and regression models represented by LR are used for survival analysis. Machine learning is used to assist decision-making in clinical studies of various cancer types (including lung cancer) and can be used with a combination of various other analysis techniques (Huang et al., 2020). NSCLC related candidate RNAs with high rankings can be derived from the RNA-seq data using pre-processing techniques. In addition, by analyzing expression differences, normal and abnormal lung adenocarcinoma (LUAD) patient groups can be comparatively analyzed. For example, in (Cai et al., 2015; Zhou et al., 2016; Su et al., 2020) a classifier for diagnosis of LUAD using SVM is proposed where molecular markers are discovered from this classifier.

**FIGURE 3:**
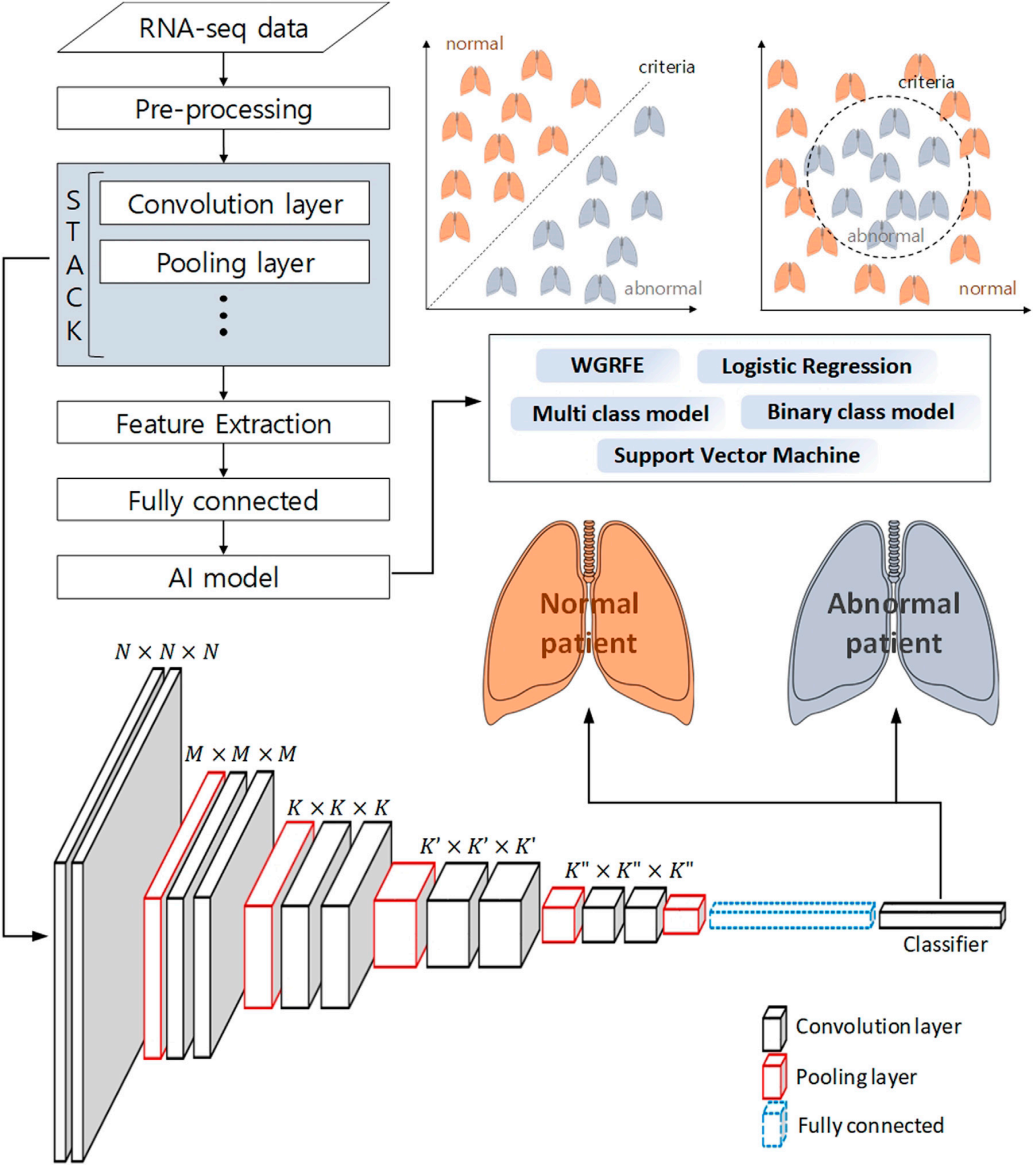
Schematic workflow of analysis to classify a normal patient from an abnormal patient using deep learning and machine learning. The pre-processed RNA-seq data is subjected to feature extraction using a neural network, and machine learning-based binary classification is performed using these features.

Machine learning techniques do not stand alone. Machine learning techniques use a combination of two or more techniques depending on their purpose. In (Afonso et al., 2019), genes were selected using the whole gene recursive feature elimination (WGRFE) technique and then subtypes of NSCLC were classified using SVM. If these ensembled models are properly combined, more accurate results can be obtained. As shown in Figure 3, by applying a classification model after analyzing with statistical analysis or deep learning tools, modules such as WGRFE and SVM can be applied in the last analyzing stage to assist the final criterion decision making. The results show that the performance can be significantly improved when machine learning techniques are used in conjunction with other analysis techniques. After pre-processing the RNA-seq data to extract the features, various techniques (e.g., statistical tools and neural networks) can be applied. The next technique to apply depends on the analysis target and the extracted features, where Figure 3 can be used to help choose the final algorithm needed to distinguish the abnormal and normal status as well as the level of difference.

### 3.3 Deep learning

Deep learning based RNA-seq analysis has been in the spotlight recently. Deep learning in the medical field has been mostly used for pattern recognition and medical image processing. For example, various imaging methods have been introduced to detect and analyze cell tissues (Volckmar et al., 2019). This is a representative method that can help reveal the origin of lung cancer, which applies imaging the tissue of cells. In these studies, deep learning is used to analyze the tumor’s microenvironment through comparative analysis between the cancer microenvironment and surrounding cells. In particular, deep learning CNN analysis on CT image data is widely used (Ma et al., 2013; Su et al., 2020). In addition, non-image data has been converted into image-like data such that CNN analysis can applied (Arbour et al., 2021). Recently, research on survival prognosis of cancer patients using deep learning has been actively conducted. However, deep learning models are rarely used as a stand-alone method in predicting a prognosis. The most widely used NSCLC deep learning models include Cox-nnet, DeepSurv, and AECOX, which are briefly described below.

#### 3.3.1 Cox-nnet

**FIGURE 4:**
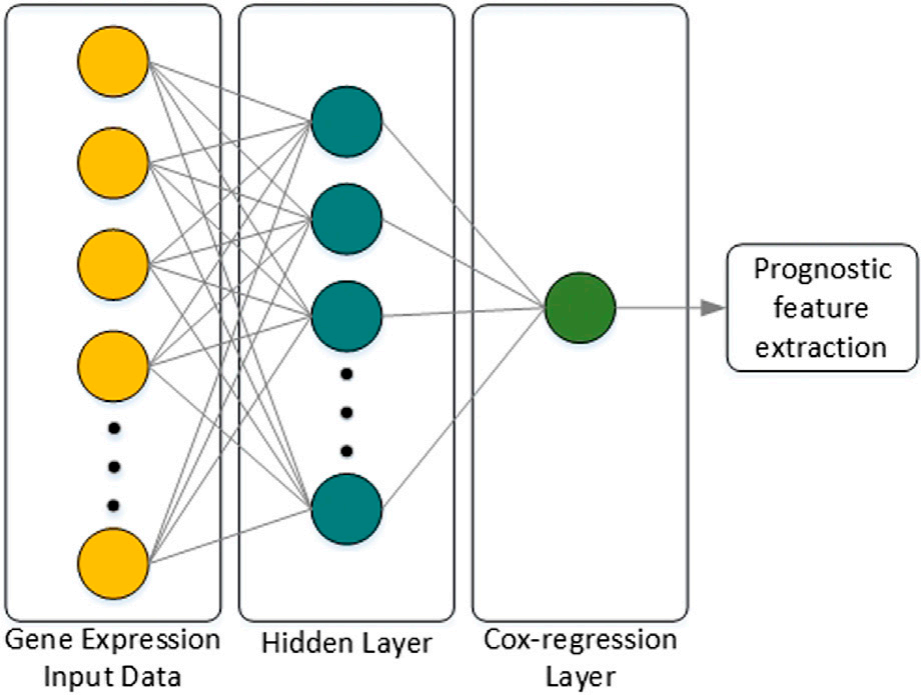
Architecture of Cox-nnet. The neural network structure is composed of the input layer, one fully connected hidden layer, and an output Cox regression layer. Finally, among many features of the patient, features related to prognosis are extracted.

Classification or prognostic prediction using the machine learning techniques described above have meaning only in the final stage analysis. Thus, research on combining the characteristics of deep neural networks (DNNs) with regression models was attempted, and an extension of the Cox-regression model using a DNN was proposed in (Travers et al., 2018), which was named Cox-nnet. The characteristics of Cox-nnet are explained in Figure 4. Cox-nnet is used to make predictions, where it was first used in cancer survival predictions. However, its simple model has a disadvantage in that it is difficult to apply when the dimension of the input data (i.e., number of input data types) increases.

#### 3.3.2 DeepSurv

DeepSurv is a multilayer perceptron model which consists of hidden layers consisting of fully-connected non-linear activation functions similar to the Faraggi-simon network consisting of a single hidden layer with two or three nodes (Faraggi and Simon, 1995). DeepSurv uses a non-linear proportional hazard model, which uses a neural network inside a Cox hazard model. DeepSurv includes one or more hidden layers, weight decay regulation, and activation functions such as an exponential linear unit (ELU) or a rectified linear unit. The DeepSurv performance can be improved by adding hidden layers to form a DNN so that the covariates of the first hidden layer of the DNN are used as input to the Cox proportional hazard model. The output of the DNN can be made to be a single node that estimates the hazard function *H*_*θ*_(*x*) parameter based on the DNN weight *θ* (Katzman et al., 2018). DeepSurv can adjust the spacing of the non-linear model distributions by adjusting the network output nodes and can predict individual non-linear distributions for a single data input. DeepSurv can be used in a variety of survival analysis applications. Examples of this approach can be found in many treatment recommendations, which is a medical application that provides treatment recommendations based on a set of patient observations.

#### 3.3.3 DCNet

**FIGURE 5:**
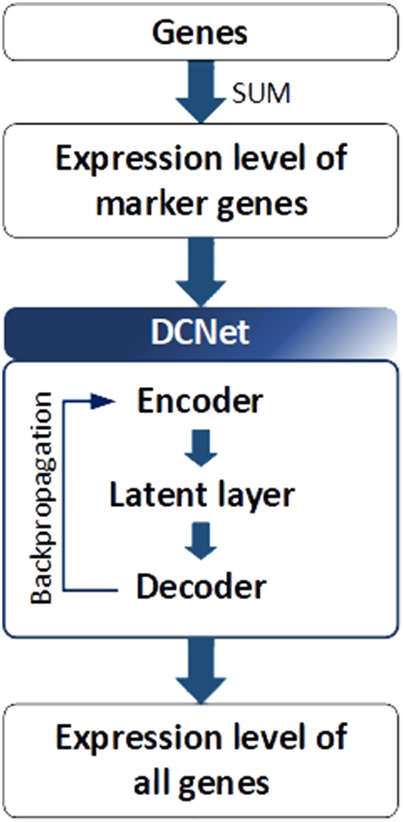
Architecture of DCNet. Gene expression levels were considered to be input and output neurons in DCNet. The expression levels of marker genes and all genes are used as input and output neurons, respectively. Finally, the encoder layer and latent layer of the neural network are transferred, and the activation value of the latent layer indicates the abundance ratio of cells.

DCNet is an autoencoder-based deep learning model that predicts about 400 cell types from a bulk RNA-seq dataset and discovers marker genes (Wang et al., 2022). As presented in Figure 5, the DCNet model consists of a total of three layers: an input layer corresponding to the marker gene, a hidden layer represented by the cell type, and an output layer composed of TCGA gene data. Therefore, it is possible to identify the relationship between a marker gene and a cell and identify a TME-specific biomarker through DCNet. DCNet can also be used for the purpose of classifying cancer subtypes. However, as in other deep learning models, the lack of data greatly affects the final performance, so to prevent this, oversampling techniques were introduced to solve the class label imbalance problem. In order to use the deep learning model effectively, a sufficient amount of training data must be secured. Then the insufficient part can be solved by using fine-tuning techniques to update the weights of the network. The DCNet model is meaningful in that it improves robustness and stability by applying a deep learning-based framework to RNA-seq research that uses simple machine learning or simple statical analysis techniques.

## 4 Conclusion

**FIGURE 6:**
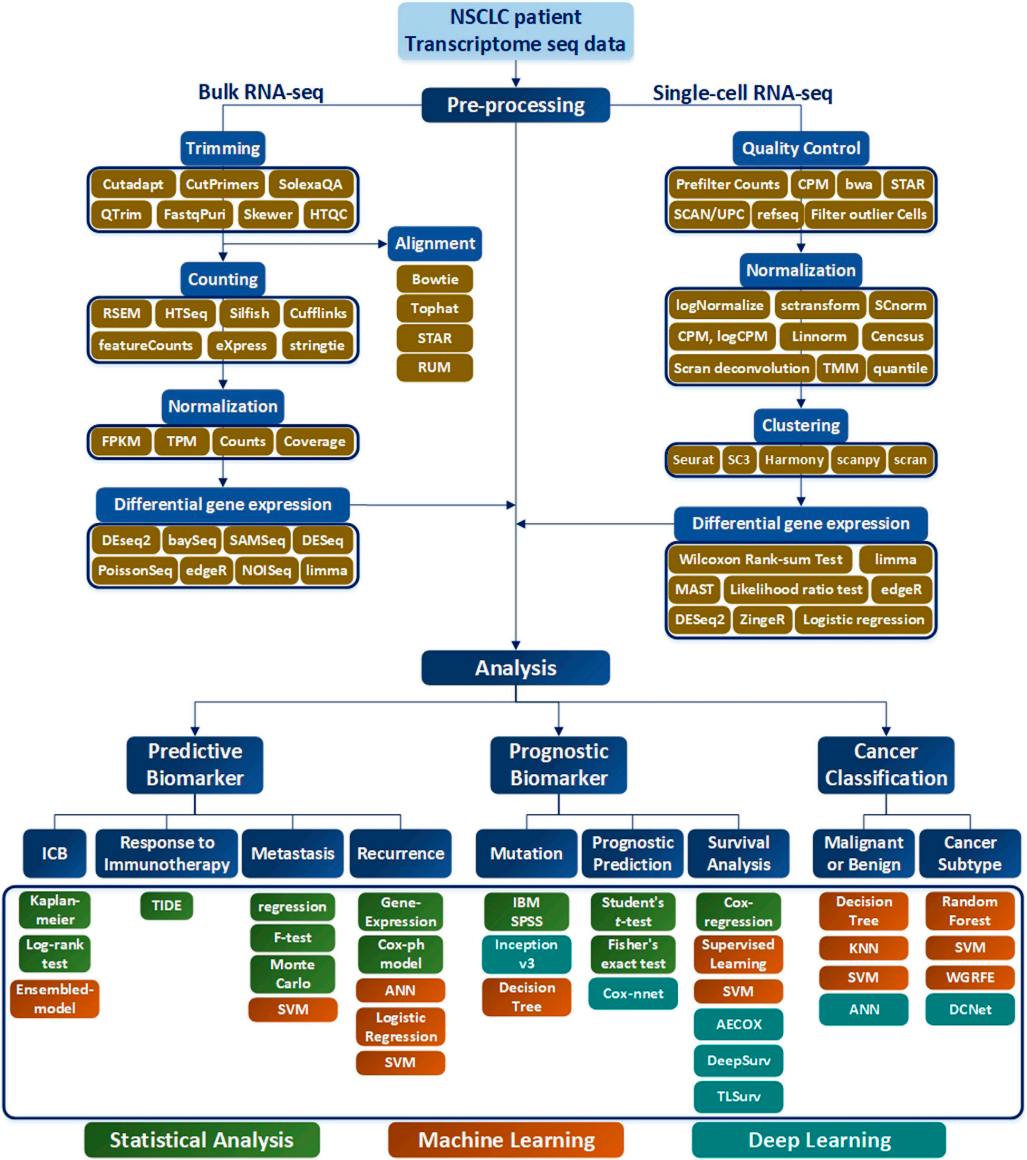
RNA-seq workflow of how to preprocess data and conduct analysis. The preprocessing section is divided into bulk RNA-seq and single-cell RNA-seq, where each require quality control. In addition, differential gene expressions are derived to obtain data for analysis. In the Analysis section, the target is classified into Predictive Biomarker, Prognostic Biomarker, and Cancer Classification, and analysis methods are sub-classified according to each purpose. In addition, the necessary tools according to the data type are indicated in green for Statistical Analysis, orange for the Machine Learning techniques, and blue for the me`thods using Deep Learning.

Selection of a RNA-seq method to apply to NSCLC data depends on the target of the analysis type, which may not be easy to select. To assist this process, in this paper, various analysis methods depending on the objective of RNA-seq are summarized in Table 1, and a methodological approach guideline is presented in Figure 6. For RNA-seq analysis of NSCLC patients, separate pipelines must be used by dividing bulk RNA-seq and single-cell RNA-seq. There is no big difference between bulk RNA-seq and single-cell RNA-seq in the basic principle of analyzing RNA-seq. However, whereas bulk RNA-seq analyzes the average value of whole cells, single-cell RNA-seq outlines each cell separately and analyzes the average value of each cell type. As shown in Figure 6, trimming to remove contamination or low-quality data and subsequent quantification are performed using various counting tools such as HTSeq. The sequence alignment process is preemptively performed. Then the pre-processing of the bulk RNA-seq can be completed by using DEseq2 or Limma to check differential gene expressions. In single-cell RNA-seq, the quality control process is carried out in units of 1 cell, where the information obtained from several cells is well classified, the similar cells are collected, and the clustering process is added, in which groups of similar cells are grouped and analyzed. Differential gene expression analysis is also performed in single-cell RNA-seq, and various tools such as MAST are used in addition to DESeq2. One effective way to analyze the subtypes of NSCLC patients would be to first cluster the NSCLC patient data corresponding to each subtype and then divide the RNA-seq data into different data sets. Using this as the input data, a classification scheme can be selected based on the analysis objective using Figure 6, and the data can be analyzed according to the number of classes. As a result, RNA that affects the subtype of the tumor can be extracted by sharp value-based ranking.

This paper investigates various AI analysis methods of RNA-seq research and provides guidance on how to apply this in NSCLC analysis and predictions. Although there are many papers on this area, there is no known single dominant method on how to apply systematic AI learning technologies in analyzing NSCLC RNA-seq data. The best way to obtain the most accurate analysis result is to select the research goal and the corresponding model properly, in which Table 1 and Figure 6 can provide some guidance. Although this paper is limited to NSCLC patients, it can be applied to other cancer types, such as breast cancer or colorectal cancer, which will be the focus of future research.
